# The *Solanum demissumR8* late blight resistance gene is an *Sw-5* homologue that has been deployed worldwide in late blight resistant varieties

**DOI:** 10.1007/s00122-016-2740-0

**Published:** 2016-06-17

**Authors:** Jack H. Vossen, Gert van Arkel, Marjan Bergervoet, Kwang-Ryong Jo, Evert Jacobsen, Richard G. F. Visser

**Affiliations:** Wageningen UR Plant Breeding, Wageningen University and Research, P.O. Box 386, 6700 AJ Wageningen, The Netherlands

**Keywords:** *Phytophthora infestans*, Potato late blight, Disease resistance gene, Cisgenesis, NB-LRR

## Abstract

*****Key message***:**

**The potato late blight resistance gene*R8*has been cloned.*R8*is found in five late blight resistant varieties deployed in three different continents. R8 recognises Avr8 and is homologous to the NB-LRR protein Sw-5 from tomato.**

**Abstract:**

The broad spectrum late blight resistance gene *R8* from *Solanum demissum* was cloned based on a previously published coarse map position on the lower arm of chromosome IX. Fine mapping in a recombinant population and bacterial artificial chromosome (BAC) library screening resulted in a BAC contig spanning 170 kb of the R8 haplotype. Sequencing revealed a cluster of at least ten *R* gene analogues (RGAs). The seven RGAs in the genetic window were subcloned for complementation analysis. Only one RGA provided late blight resistance and caused recognition of Avr8. From these results, it was concluded that the newly cloned resistance gene was indeed R8. R8 encodes a typical intracellular immune receptor with an N-terminal coiled coil, a central nucleotide binding site and 13 C-terminal leucine rich repeats. Phylogenetic analysis of a set of representative *Solanaceae* R proteins shows that R8 resides in a clearly distinct clade together with the Sw-5 tospovirus R protein from tomato. It was found that the *R8* gene is present in late blight resistant potato varieties from Europe (Sarpo Mira), USA (Jacqueline Lee, Missaukee) and China (PB-06, S-60). Indeed, when tested under field conditions, *R8* transgenic potato plants showed broad spectrum resistance to the current late blight population in the Netherlands, similar to Sarpo Mira.

**Electronic supplementary material:**

The online version of this article (doi:10.1007/s00122-016-2740-0) contains supplementary material, which is available to authorized users.

## Introduction

Genetic resistance against pests and diseases is the most sustainable crop protection strategy (Michelmore et al. 2013) and has already provided durable solutions in many different agrosystems. Potato late blight, caused by the oomycete *Phytophthora infestans*, is still a serious problem for one of the major food crops in this world. Despite the fact that genetic resistance to late blight is amply available in the potato germplasm (Vleeshouwers et al. 2011; Vossen et al. 2014), it is deployed in potato varieties only to a limited extent. Limiting factors in late blight resistance breeding are the long breeding cycles and the highly heterozygous tetraploid genome. Also, *P. infestans* is notorious for its short asexual spore cycles, allowing mitotic mutations, and sexual generation which allows rapid genetic recombination in many regions of the world. To achieve durable resistance to late blight, multiple resistance (*R*) genes must be introduced in varieties to provide incremental and insurmountable hurdles for *P. infestans,* thereby further delaying the breeding process. So, the rigidity of the potato genome and the flexibility of the *P. infestans* genome have so far prevented the large-scale use of resistant varieties. Sarpo Mira is a variety that shows durable resistance to the current *P. infestans* population (Lees et al. 2012), but this variety is not widely grown because agricultural and industrial processing characteristics of late blight susceptible varieties like Bintje and Russet Burbank are preferred. Improvement of established varieties through genetic modification is therefore an obvious approach; especially the introduction of natural genes from crossable species, known as cisgenes, is associated with low risks and is preferred by consumers (Eurobarometer 2010; Devos et al. 2014). In the last 10 years, the cloning of at least eight cisgenic late blight *R* genes has been reported and many more are available from the germplasm (Rodewald and Trognitz 2013). The simultaneous introduction of multiple cisgenes causing late blight resistance has been shown to be a feasible and highly efficient approach (Zhu et al. 2012; Jo et al. 2014). For a viable cisgenic late blight breeding approach, many cloned broad-spectrum *R* genes must be available. The potato late blight differential Ma*R8* is considered a valuable late blight resistance source, because virulence towards Ma*R8* is found only with low frequency. The gene responsible for Ma*R8* resistance is referred to as *R8* (Jo et al. 2011; Kim et al. 2012)*. R8* has the same map position and recognition specificity as *Rpi*-*smira2* (Jo 2013), the main determinant of the resistance in the potato variety Sarpo Mira (Rietman et al. 2012) that has remained resistant already for several years. Also, the late blight *R* gene from the variety Jacqueline Lee is located at a similar genetic position (Massa et al. 2015). Here, we report the cloning of the *R8* gene through a map-based cloning approach which includes a fine mapping, BAC landing, BAC walking, candidate cloning and complementation analysis. We show that *R8* encodes a CC-NB-LRR protein with 89 % identity to Sw-5, a tomato spotted wilt virus resistance R protein.

## Materials and methods

### Plant material

The potato differential plant Ma*R8,* corresponding to plant 2424a(5) described by Black et al. (1953), was used for bacterial artificial chromosome (BAC) library construction. MaR8*Concurrent population (code 3020) was used for genetic mapping. These plant materials and cv Desiree, which was used for transformation, were maintained in vitro at Wageningen UR Plant Breeding. *Nicotiana benthamiana* was maintained as seed stock. PB-06 (387132.2*387170.9), S-60 (393075.54* 391679.7), and C-88 (Li et al. 2010) were maintained at Yunan University. Jacqueline Lee (Tollocan*Chaleur; Douches et al. 2001) and Missaukee (Tollocan*NY88; Douches et al. 2009) were maintained at Michigan State University. Isolated DNA was analysed in Wageningen.

### Bacterial artificial chromosome library construction and screening

A first BAC library was produced by mechanical shearing of Ma*R8* genomic DNA and ligation of high molecular weight fragments into pCC1 at RxBiosciences (Gaithersburg, MD, USA). This first BAC library consisted of 768 simple pools of 200 individual BAC clones. Simple pools were stored at −80 °C. The average insert size was ~55 kb, resulting in a 2.5* coverage of a haploid genome. A second BAC library was produced by Bio S&T (Saint-Laurent, Montreal, Canada). Ma*R8* genomic DNA was fragmented by partial digestion with *Hin*dIII. Size-selected fragments were cloned into pIndigoBAC-5. The average insert size was ~100 kb (Fig. S1). The 750 simple pools of 400 individual BACs, representing a 10* coverage of the haploid genome, were stored at −80 °C. Markers described in Table S1 were used to screen the BAC libraries. Bacterial suspensions of positive pools were diluted and plated on LB agar plates containing chloramphenicol (12.5 μg ml^−1^). After determining the bacterial titre of a positive pool, 2 × 96 subpools containing 50 individuals each were grown for 8 h in deepwell blocks. After culture, PCR-positive subpools were plated on LB plates containing chloramphenicol and individual colonies were picked into 96 flat-bottom microtitre plates. Positive BAC clones were subsequently identified by a third round of PCR screening.

### DNA sequencing and bioinformatics analysis

BAC clone sequencing was carried out using a shotgun strategy. Fragmentation, library production, 454 sequencing and contig assembly were performed at Macrogen (Seoul, Korea). BAC n2A2 was sequenced using PACBIO (GATC, Germany). Gene structures were predicted using FGENESH2.6 (Softberry) and protein sequences were deduced by translation of ORF using the standard genetic code. Multiple sequence alignments and phylogenetic analyses were conducted using CLUSTALX 1.81 (Thompson et al. 1997) available in the MegAlign Lasergene 9.0 software package (DNASTAR Inc., USA).

### (Sub)cloning of candidate genes

**Fig. 1:**
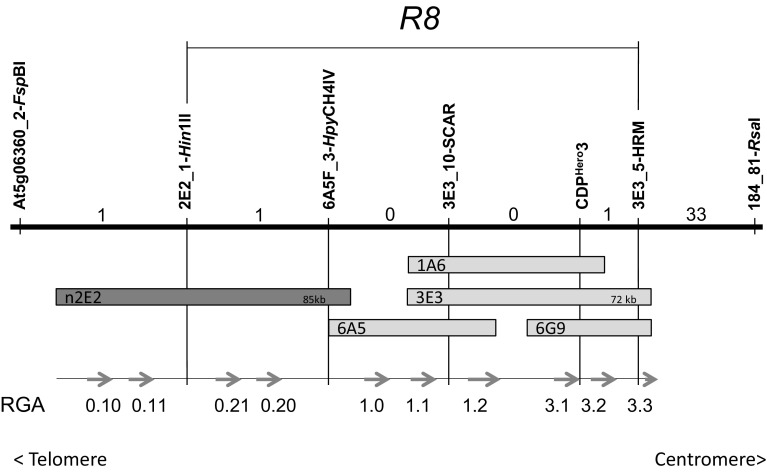
Genetic and physical map of *R8* on the bottom end of chromosome 9. The upper part of this figure represents a genetic map. Markers of different origin were mapped in an F1 recombinant population of 1670 individuals and their relative positions are indicated by *vertical lines*. The number of recombinants between the markers is indicated by numbers between the *vertical lines*. The *horizontal line* marked by *R8* indicates the genetic mapping interval for *R8*. The lower part of this figure represents a physical map. Using molecular markers, BAC clones were identified from BAC libraries derived from Ma*R8*. The *grey bars* represent the individual BAC clones. *Vertical lines* indicate the connection between the physical and genetic maps. *Arrows* indicate the position and orientations of resistance gene analogues (RGA0.10-RGA3.3) on the physical map. RGA0.20 is the functional *R8* gene

Primers were designed for subcloning RGA0.20-3.2 (Fig. 1) using primer select from the Lasergene 9.0 software package (DNASTAR Inc., USA) and were extended at the 5′ end with recognition sites for eight cutter restriction enzymes (Table S2). Long-range PCR amplifications were performed using Phusion^®^ High-Fidelity DNA Polymerase (New England Biolabs, Ipswich, USA). Reaction conditions were 98 °C for 30 s, followed by 24 cycles of 98 °C for 10 s, 62–65 °C for 30 s, 72 °C for 5.5 min and a final extension time of 15 min at 72 °C. BAC clones 3E3, 6A5, or n2E2 were used as templates. The resulting PCR products were subjected to G50 Sephadex purification using Illustra MicroSpin columns (GE Healthcare) followed by ligation to the PCR-BluntII-Topo vector using the Zero Blunt Topo PCR Cloning Kit (Invitrogen). The ligation products were transformed to ElectroMAX *E. coli* DH10B competent cells (Life technologies, Paisley, UK). The inserts of PCR-positive colonies were sequenced using a primer walking strategy (700 × 700 bp) to confirm that no mutations were introduced. The purified PCR-BluntII-Topo clones were digested with *Asc*I and *Sbf*I, or with *Xma*I and *Sbf*I which were present in the 5′ extensions of the primer (Table S2), Sticky ends were subsequently dephosphorylated using TSAP (Pomega) and all enzymes were heat inactivated. The digestion mix was ligated to the *Asc*I and *Sbf*I or *Xma*I and *Sbf*I sites of the binary vector pBINPLUS–PASSA (Jo et al. 2016), which is a modified version of pBINPLUS containing an eight cutter multiple cloning site. For *R8* allele mining in potato varieties, primers R8-AbsI-F and R8-SrfI-R (Table S2) were used according to the same procedure as described above. Instead of BAC DNA, the genomic DNA of the respective varieties was used as a template.

### Late blight resistance tests

*Phytophthora infestans* isolate IPO-C (race 1, 2, 3, 4, 5, 6, 7, 10, 11) was used for all late blight assays in this report. The only exception was the late blight field trial in 2014, which was a result of natural *P. infestans* infection. Field trials for the transgenic Desiree plants and non-transgenic recombinant plants from the extended 3020 population were performed in four replicates per genotype as described earlier (Jo et al. 2011) in the growing season of 2013 and 2014 in Wageningen, The Netherlands. Disease assessments were made by observing late blight lesions in the leaves at weekly intervals after inoculation (July 1st 2013) or after the first late blight symptoms were visible (June 23rd 2014). The observations were stopped in the second week of August. For whole plant climate chamber assays, in vitro plantlets of transgenic Desiree plants or recombinants from population 3020 were planted in pots and grown in the greenhouse at 22 °C with a 10 h day/14 h night photoperiod and a relative humidity of 70–80 %. One month after potting the plants, they were transferred into a growth chamber and inoculated. The inoculum was prepared essentially as described (Vleeshouwers et al. 1999) and plants were inoculated by placing four 10 µl droplets of inoculum (5 × 10^4^ zoospores/ml) on the adaxial side of the leaf. Three leaves per plant and two replicate plants per genotype were inoculated. The inoculated plants were kept for 7 days in a cooled climate chamber at 15 °C and 100 % humidity with a photoperiod of 16 h/8 h day/night. Late blight levels could be classified into three groups, resistant [no symptoms, limited hypersensitive response (HR)], intermediate resistance (large HR lesions or spreading HR lesions without sporulation) or susceptible (sporulating lesions). Genotypes classified in the resistant group or intermediate resistant group in climate chamber assays did not show significant late blight lesions until the end of the field trial experiments and could easily be distinguished from the susceptible group.

### *Agrobacterium*-mediated transient co-expression in *N. benthamiana*

Binary plasmids harbouring RGAs or Avr8 (Jo 2013) were transformed to *A. tumefaciens* strain AGL1 with an additional plasmid-borne copy of VirG (van der Fits et al. 2000). Two leaves per plant and three replicates of 4-week-old *N. benthamiana* seedlings were agroinfiltrated. A mixture of *R3b* and *Avr3b* (Li et al. 2011) was used as the positive control and empty pBINPLUS was used as a negative control*. A. tumefaciens* strains from frozen glycerol stocks were grown overnight at 28 °C in 3 ml of LB medium supplemented with appropriate antibiotics. The next day, these cultures were used to inoculate 15 ml of YEB medium (5 g beef extract, 5 g bacteriological peptone, 5 g sucrose, 1 g yeast extract, 2 ml 1 M MgSO_4_ in 1 l of milliQ water) supplemented with antibiotics, 10 µl/l of 200 mM acetosyringone and 1000 µl/l of 1 M MES. On the third day, the cells were harvested and resuspended to a final OD_600_ of 0.2 in MMA (20 g sucrose, 5 g MS salts and 1.95 g MES in 1 l of distilled water, adjusted to pH 5.6 with KOH) supplemented with 1 ml/l of 200 mM acetosyringone in DMSO. Responses were scored 3–4 days after infiltration.

### Transformation of potato

Binary plasmids harbouring RGAs were transferred to *A. tumefaciens* strain AGL1 containing the helper plasmid pVirG (van der Fits et al. 2000). The stability of these clones in *Agrobacterium* was tested and overnight cultures of the transformed *A. tumefaciens* strain were used to transform the susceptible cultivar Desiree (Heeres et al. 2002). The kanamycin-resistant regenerants (transgenic events) were analysed by PCR to determine the presence of the desired *R8* gene. Two or four plants per transgenic event were transferred to the greenhouse for climate chamber assays or for planting in the field, respectively.

## Results

### *R8* fine mapping

To fine map *R8*, molecular markers were required to perform a recombinant screen in the F1 population 3020 (MaR8*Concurrent). *R8* is located at the bottom end of chromosome 9, flanked by Tm-2^2^-like CDP markers at the proximal side and by Sw-5 CDP markers on the distal side (Jo et al. 2011). The CDP markers were not suitable for high-throughput recombinant screens and simple PCR markers needed to be developed. On the proximal side, marker 184_81-*Rsa*I had been described before but a marker on the distal side of *R8* remained to be developed. Screening of the tomato marker database revealed marker C2_At5g06360, which is located near the telomere of Chr9. Ma*R8* and cv concurrent derived amplicons of this marker were screened for cleaved amplified polymorphisms linked to resistance and this resulted in marker At5g06360_2-*Fsp*BI. Population 3020 was expanded to 1720 individuals, and recombinants between markers At5g06360_2-*Fsp*BI and 184_81-*Rsa*I (Fig. 1) were screened for. In total, 36 recombinants were found and their resistance phenotype was determined in a whole plant late blight assay in a climate chamber. Marker CDP^Hero^3, which was identified previously (Jo et al. 2011), was still fully linked to resistance in this expanded population. Two recombinants were found between At5g06360_2-*Fsp*BI and CDP^Hero^3, while 34 recombinants were found between 184_81-*Rsa*I and CDP^Hero^3 (Fig. 1).

### BAC landing and BAC walking

A first, the BAC library was constructed from the genomic DNA of Ma*R8* plants. The library was screened using marker CDP^Hero^3, and BAC clones 1A6, 3E3 and 6G9 were identified. The insert of 3E3 was sequenced and revealed the presence of four complete (RGA1.1, 1.2, 3.1 and 3.2) and one truncated *R* gene analogue (RGA3.3). The newly obtained sequences were used for new marker development. A screen for markers in the intergenic regions successfully identified two polymorphic markers named 3E3_5-HRM and 3E3_10-SCAR. Mapping of the new markers revealed no recombinants at the left end of the BAC, indicating that RGAs 1.0, 1.1, 3.1, 3.2 and so far unidentified additional RGAs could be *R8* candidates. The right end of BAC 3E3 fell outside the mapping interval, excluding RGA3.3 as an *R8* candidate (Fig. 1). To close the genetic window, marker 3E3_10-SCAR was used for screening the BAC library which resulted in the isolation of the BAC clone 6A5 (Fig. 1). Sequence analysis revealed one additional complete RGA (RGA1.0). A marker developed on the 6A5 BAC end (6A5F_3-HpyCH4IV) still did not show any recombinants with *R8* resistance, so the genetic window was not closed yet. Marker 6A5F_3-HpyCH4IV was used to screen the BAC library, but unfortunately no new positive BACs were identified. A new BAC library was generated using a different genome fragmentation method (partial restriction enzyme digestion instead of mechanical shearing that was used in constructing the first BAC library). Screening of the new library identified the BAC clone n2E2. Sequence analysis revealed the presence of four additional complete RGAs (0.10, 0.11, 0.20, and 0.21.). A screen for markers in the intergenic regions revealed marker 2E2_1-Hin1II. One recombinant was found between this marker and the late blight resistance and it was concluded that the genetic interval was now closed between markers 2E2_1-Hin1II and 3E3_5-HRM. All together, a genomic region of 170 kb (GenBank accession number KU530153) containing a cluster of ten paralogous RGA sequences was found. All sequences had high homology to *Sw*-*5*, an *R* gene from tomato that provides resistance to tomato spotted wilt virus (Brommonschenkel et al. 2000).

### *R8* candidate cloning and complementation analysis

**Fig. 2:**
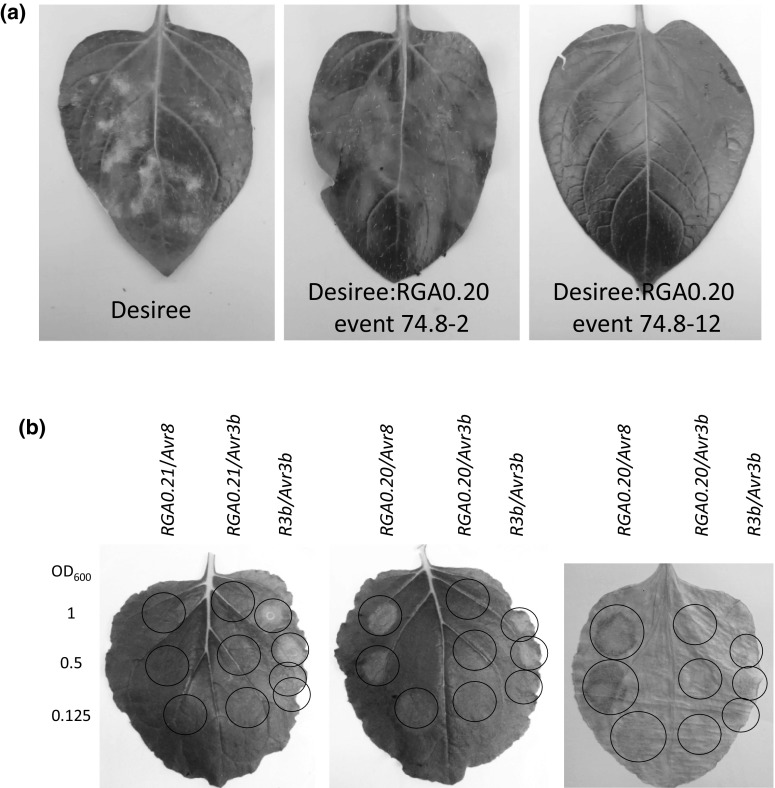
*R8* complementation analysis. **a** C. Non-transgenic Desiree, after inoculation with IPO-C in a whole plant assay, was fully susceptible as apparent by the sporulating lesions (*white* appearance in the *left* image). Transgenic Desiree events containing RGA0.20 were no longer susceptible, as phenotypes ranged from intermediate resistance (like in event A74.8-2 where huge HR lesions are observed without any spores), to full resistance (like in event A74.8-12 where no or only very small HR lesions were found). **b** Co-expression of RGA0.20 and *Avr8* in *N. benthamiana* results in a hypersensitive response. 1:1 mixing ratios of RGA0.20 and *Avr8* were diluted to OD_600_ = 1, 0.5, or 0.25. 1:1 mixes of *R3b* and *Avr3b* were used as positive controls. HR was not observed upon co-expression of Avr8 and any of the other candidate genes (RGA0.21 is here provided as an example). As the HR could not be visualised with high contrast, we dried out the leaves. As shown in the *right* image, specific discoloration at the site of co-expression of *R8* and *Avr8* is now clearly visible

The seven RGAs in the genetic window were subcloned in the binary vector pBINPLUS–PASSA for *Agrobacterium*-mediated transformation of plants. Stable transgenic plants of the susceptible potato variety Desiree were produced and 10–47 events per construct were selected (Table 1). Six out of seven constructs produced only transformation events that were susceptible to *P. infestans* isolate IPO-C. Eight out of 47 events transformed with RGA0.20 were susceptible, while 39 events showed intermediate to strong late blight resistance in a whole plant assay in climate chambers (Fig. 2a). PCR analysis revealed that the eight susceptible events contained only partial inserts of the T-DNA. RGA0.20 was therefore denoted as a strong *R8* candidate. This idea was confirmed when the RGAs were co-expressed with *Avr8* in the *N. benthamiana*. Only RGA0.20 induced a hypersensitive response (HR) when co-infiltrated with *Avr8* (Fig. 2b). The observed HR was a result of specific recognition, since co-infiltration of *Avr3b* with RGA0.20 did not result in an HR. Co-expression of RGA0.20 with other known Avr genes (Avr2, Avr3a and Avrvnt1) also did not result in an HR (data not shown). From these results, we conclude that RGA0.20 is *R8*.

**Table 1:** Complementation analysis of *R8* resistance in Desiree

Construct	Avr8 response	Climate chamber whole plant assay^a^			Field trial 2014^b^	Field trial 2015^b^	Field trial 2015^b^
		S	IR	R			
					(ex vitro)	(ex vitro)	(tuber)
RGA3.2	−	12/12	0/12	0/12	nd	nd	nd
RGA3.1	−	10/10	0/10	0/10	nd	nd	nd
RGA1.2	−	10/10	0/10	0/10	nd	nd	nd
RGA1.1	−	10/10	0/10	0/10	nd	nd	nd
RGA1.0	−	10/10	0/10	0/10	nd	nd	nd
RGA0.20	+	8/47	14/47	25/47	39/47	14/14	13/13
RGA0.21	−	15/15	0/15	0/15	nd	nd	nd
Vector only	−	12/12	0/15	0/15	0/12	0/4	nd

“−” no hypersensitive response observed upon co-agroinfiltration in *N. benthamiana.* “+” a hypersensitive response observed upon co-agroinfiltration in *N. benthamiana*

*nd* not determined

^a^Number S(usceptible), I(ntermediate) R(esistant), or R(esistant) events over the number of tested events

^b^Number of resistant events over the number of tested events

**Fig. 3:**
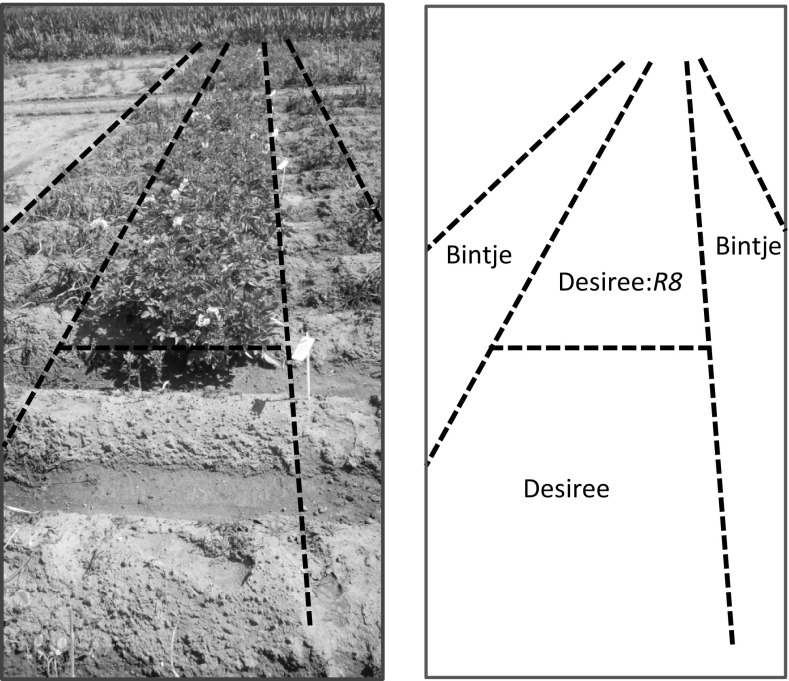
*R8*-mediated broad-spectrum resistance in field trials. Bintje spreader rows were derived from seed tubers. Desiree and Desiree transgenics were planted from in vitro culture. For this reason, at the onset of the natural late blight epidemic, the Bintje plants were much taller than the Desiree and *R8* transgenics (Desiree:*R8*). This explains the difference in the height of the deceased Bintje and Desiree plants, while the *R8* transgenics kept growing

Not only the molecular recognition pattern of *R8* was matched, but also the broad resistance spectrum of *R8* against the current *P. infestans* population was maintained. 39 transgenic events provided excellent late blight resistance to natural late blight infection in 2014 in a field trial in Wageningen (Table 1; Fig. 3). Events that showed resistance in 2014 were planted again in the field in 2015. Fourteen events were propagated in vitro (referred to as ex vitro) and 13 of these events were also propagated using seed tubers. All plants were fully resistant, showing that events can be selected that stably express the resistance after clonal propagation.

### *R8* sequence annotation

**Fig. 4:**
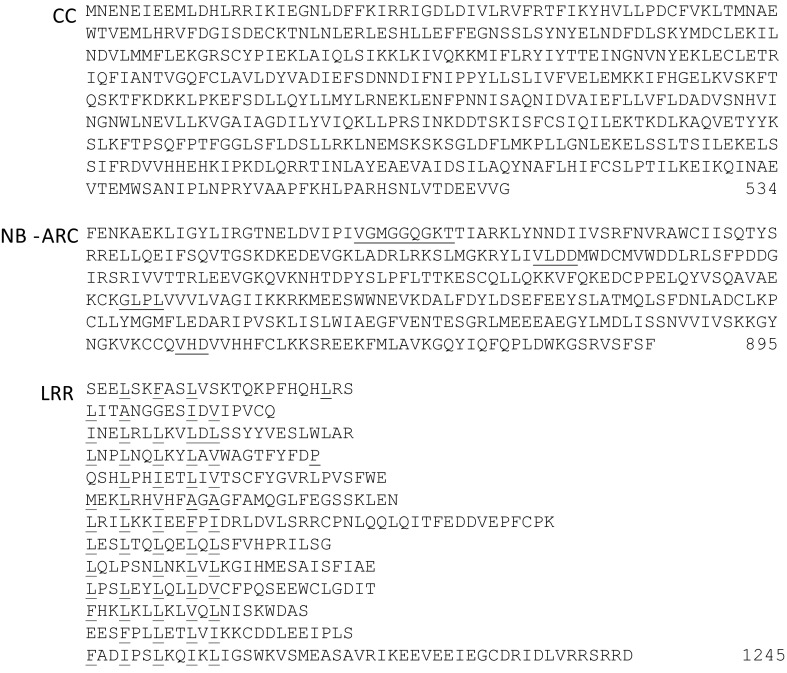
Amino acid sequence architecture of the R8 protein. Coiled coil (CC) forming amino acid stretches were found in the N-terminal domain (*red* *font*) as determined using the COILS algorithm (Lupas et al. 1991; window = 14 aa, threshold >0.1). In the central region Nucleotide binding Apaf-1 *R* gene and CED4 homology (NB-ARC; van der Biezen and Jones 1998) can be distinguished (*underlined*). In the C-terminal region, leucine-rich repeat (LRR) regions matching the consensus lxxlxxlxxlxl can be distinguished (*underlined red font*). *Figures* indicate the position of the preceding amino acid residue in the protein

**Fig. 5:**
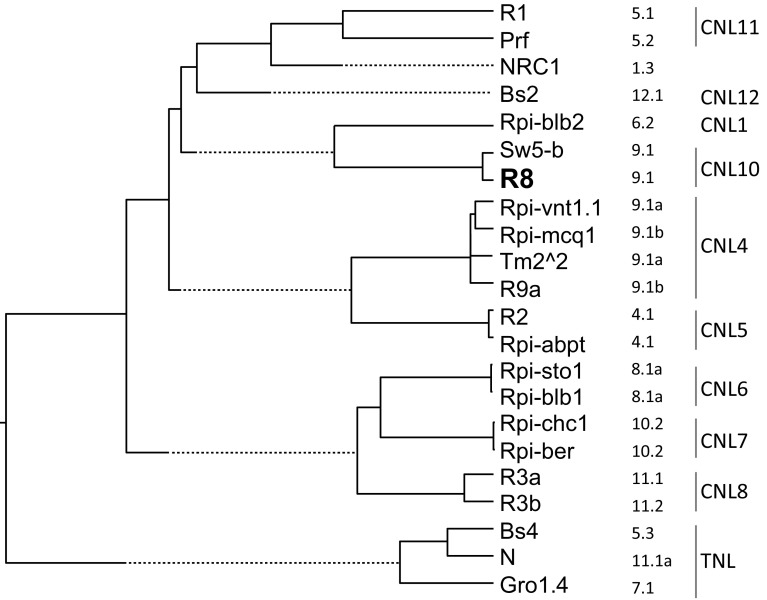
Phylogenetic analysis of R8 and the other R proteins from Solanaceae. The *column on the* *right* of the protein names contains the genetic location of the encoding genes (chromosome, and RGA cluster number; Vossen et al. 2013). Clades observed in this tree match the CC-NB-LRR (CNL) and TIR-NB-LRR (TNL) groups defined previously (*extreme* *right column*; Andolfo et al. 2014). R1: Q8W1E0; Prf: AAC49408; NRC1: NP_001234202; BS2: AAF09256; Rpi-blb2: AAZ95005; Sw5-b: AAG31014; Rpi-vnt1.1: ACJ66594; Rpi-mcq1: Jones et al. (2009); Tm2^2: AAQ10736; R9a: de Vetten et al. (2011); R2: ACU65456; Rpi-abpt: ACU65455; Rpi-blb1: Q7XBQ9; Rpi-chc1, Rpi-ber: Vossen et al. (2010); R3a: AAW48299; R3b: AEC47890; BS4: XP_010320695; N: Q40392; Gro1.4: AAP44390;)

The binary vector containing *R8* that was used for complementation studies had an insert of 7011 bp. The 5′ untranslated region of 1680 bp encompasses a functional promotor and is followed by a single open reading frame of 3738 bp, encoding a 1245 aa R8 protein and a stop codon, which is followed by a 1594 bp 3′ untranslated region that encompasses a functional transcriptional terminator. The encoded R8 protein showed a tripartite domain structure, which is typical for intracellular plant disease resistance proteins. An N-terminus with predicted coiled coil (CC) structures, a central nucleotide binding (NB) and a set of 13 C-terminal leucine-rich repeats (LRR) were found (Fig. 4). When the R8 protein sequence was aligned with known R proteins from Solanaceae and phylogenetic analysis was performed, it was found that R8 constitutes a distinct clade with the tomato Sw-5 protein (Brommonschenkel et al. 2000), which provides resistance to tomato spotted wilt virus (Fig. 5). This clade matches the CC-NB-LRR group 10 (CNL10) as defined by Andolfo et al. (2014). Sw-5 and R8 shared 83.3 % identity over the entire protein (81.9, 89.8 and 77.5 % of identity in the CC, NB-ARC and LRR regions, respectively), while identity over the entire protein to other R proteins (NRC1, R1, Rpi-blb2, Prf, Rpi-chc1, Rpi-ber, Rpi-vnt1.1, Rpi-blb1, Bs2, Bs4, Gro1.4, R2, R3a, R3b, Tm2^2, R9a, Rpi-mcq1 and N) ranged between 26.1 %, in the case of *Rpi*-*blb2*, and 15 % in the case of Gro1.4 (Table S3).

### *R8* in breeding germplasm

Two reports are available that show the presence of late blight *R* genes at the same genetic location as *R8* in commercial varieties (Jo 2013; Massa et al. 2015). To test if these *R* genes were related to *R8*, we pursued an allele mining approach. Genomic DNA samples of Sarpo Mira, Jacqueline Lee and its half-sib Missaukee, and also from Chinese varieties (C-88, PB-06, and S-60) with reported late blight resistance, were used as template in a long-range PCR using primers encompassing the complete *R8* gene. The susceptible variety Desiree was used as a negative control. Sarpo Mira, Jacqueline Lee, Missaukee, PB-06 and S-60, but not C-88 and Desiree, produced a fragment of the expected size (7 kb). Also nontemplate controls showed no amplicon, ruling out that contamination with the same template had occurred. The PCR fragments were cloned and the inserts of at least three independent *E. coli* clones per variety were sequenced. The coding sequences of all Sarpo Mira, Jacqueline Lee, Missaukee, PB-06 and S-60 amplicons were identical to the *R8* sequence. Outside the coding sequence, only one single nucleotide polymorphism was found in the 5′UTR of the Sarpo Mira allele, suggesting that the *R8* haplotypes had been recently derived from the same ancestor. The *Rpi*-*smira2* gene was previously shown to locate at the same position as *R8* and to cause AVR8 recognition. Now, we have found that Sarpo Mira contains a sequence identical to *R8*; we conclude that *R8* and *Rpi*-*smira2* are allelic.

## Discussion

### Map-based cloning of *R8*

Using a classical map-based cloning approach, we have identified the *R8* late blight resistance gene. Three rounds of BAC landing and BAC walking were required to capture the genetic window within a physical map of 170 kb. The process was highly labour intensive, as only two recombinants were found at the flanks of the 170 kb region (1 rec/85 kb). The physical distance between 3E3_5-HRM and 184_81-*Rsa*I, as compared to the DM reference genome, was only 430 kb, but in this interval 33 recombinants were found (1 rec/13 kb). It is remarkable that the recombination frequency in the subtelomeric end of the chromosome is much more higher than in the telomeric end. This might be a result of the introgression of the *R8* locus from *S. demissum*, which is less compatible for recombination with its sister chromatids from *S. tuberosum*. A recombination block because of segmental inversion is unlikely as the *S. phureja* and *S. lycopersicon* reference genomes carry similar numbers of RGAs in the same orientation as we have found (Jupe et al. 2012; Andolfo et al. 2014). A second reason for the labour intensity is that the first BAC library that was produced did not completely cover the *R8* genomic region and a new BAC library needed to be constructed. Next-generation sequencing protocols could potentially reduce the labour intensity of map-based cloning, as the tedious BAC walking steps might become redundant. Whole genome resequencing is not sufficiently powerful yet for heterozygous polyploid genomes, such as potato. The identification of *R* genes, which are notorious for the number of paralogs in a single haplotype, from whole genome sequences is a particular challenge. Complexity reduction methods such as Renseq (Jupe et al. 2012) or next gen-profiling (Vossen et al. 2013) linked to single molecule sequencing platforms currently provide the best opportunity to accelerate *R* gene cloning.

### *R8* as a member of the CNL10 group

*R8* showed 83.3 % identity to *Sw*-*5* from tomato, which is involved in the recognition of a very different pathogen, i.e. a negative strand RNA virus. Several other *R* genes have been mapped in this location, among which the potato gene *Gpa6* confers resistance to the white potato cyst nematode *Globodera pallida* (Rouppe van der Voort et al. 2000) and the potato virus Y resistance gene from *S. chacoense* (Sato et al. 2006), but none of them have been reported to be cloned and could not be confirmed as CNL10 sequences. Interestingly, an effector protein from the golden cyst nematode *Globodera rostochiensis* targets a host protein from the CNL10 family (Rehman et al. 2009). However, it remains elusive how this protein–protein interaction affects plant–pathogen interaction(s).

The finding of highly homologous *R* genes that confer resistance to very diverse pathogens is not unique. *Rx1, Gpa2* and *Bs*-*2* from the CNL2/12 group on chromosome 12 recognise bacteria, nematodes or viruses, respectively (Bendahmane et al. 1999; Tai et al. 1999; van der Vossen et al. 2000). The guard hypothesis assumes that pathogen effectors interact with virulence targets in the plant host. Perturbations of these virulence targets are sensed by NB-LRR receptors (De Wit et al. 2009). It has been speculated that *R* genes have evolved around a limited set of virulence targets or decoys that are shared by many different pathogens, thereby limiting the number of receptor molecules needed to detect the multitude of pathogen effectors (van der Hoorn and Kamoun 2008). Another explanation for the high level of homology among CNL10 members as opposed to the highly diverse recognition spectra may be found in the recognition of pathogen-derived ligands through R protein pairs (Bonardi et al. 2011; Sohn et al. 2014; Wu et al. 2016). The combination of different R protein pairs could drastically alter the recognition specificity.

The cloning of *R8* and the identification of *Avr8* (Jo 2013) now provide the tools for the comparative study of CNL10 members at the molecular and functional level. Also, it will be interesting to study how the different CNL10 proteins signal to evoke a resistance reaction and how host resistance reactions or pathogen resistance suppression mechanisms can potentially interfere.

### *R8* and durability of resistance

The role of *R8* as a late blight resistance source with high potential has been recognised already for a long time, since virulence towards *R8* is only rarely encountered in *P. infestans* populations around the world (Swiezynski et al. 2000; Haynes et al. 2002; Zhang and Kim 2007). Also in this study, we found that *R8* provides particularly strong resistance against the current late blight population in The Netherlands (Fig. 3). *R8*’s potential to contribute to durability was confirmed, since the major resistance component of the durably resistant potato variety Sarpo Mira, *Rpi*-*mira*-*2*, was allelic and identical to *R8*. Also, the R8 was found in durably resistant varieties from the USA and China.

It cannot be assessed if Sarpo Mira’s, Jacqueline Lee’s or Missaukee’s durability is associated with its relatively small acreage, or with the presence of highly complementary (*R*) genes elsewhere in their genome. Some indications about the durability potential of *R8* came from a study where plants that only contained *R8*, or *R8* in combination with multiple different *R* genes, were exposed to natural late blight infection. It was found that plants carrying only *R8* had a similar delay in the onset of late blight symptoms as Sarpo Mira (Kim et al. 2012). This study was, however, performed using plants derived from sexual crosses and inherently had divergent genetic constitutions that are renowned to severely affect the outcome of late blight resistance assays (Collins et al. 1999; Gebhardt et al. 2004). More reliable results can be obtained using plants that carry single or multiple *R* genes in isogenic genetic backgrounds. The transgenic *R8* Desiree plants presented in this study are a valuable addition to the recently presented GM differential set (Zhu et al. 2014) and can be used to monitor virulence towards *R8* in *P. infestans* populations. Despite the potentially good durability perspectives of *R8* in late blight resistance breeding, we anticipate that, upon large-scale cultivation, resistance based only on *R8* will sooner or later be overcome. It is known that the resistance levels of varieties with large acreage China gradually decline (Li et al. 2010). So, late blight resistant varieties must be deployed only in combination with a resistance management strategy. To avoid that, virulence builds up in *P. infestans* populations. Targeted biocide sprays and deployment of *R* gene stacks can be part of such management strategies (Haverkort et al. 2016). So, the efficacy and complementarity of *R8* with other *R* genes must be tested and validated. To rapidly make plants with such *R* gene combinations, again, transformation is the preferred strategy.

### *R8* and classical late blight resistance breeding

Sarpo Mira shows good resistance to late blight due to *R8* (Rietman et al. 2012), but (other) disease resistance is also encoded on unlinked genomic loci (Tomczyńska et al. 2013, 2014). However, Sarpo Mira is not deployed on a large agricultural scale, because other characteristics of this variety are suboptimal and additional breeding steps are required.

The *R8* sequence described in this study is a useful tool to design broadly applicable molecular markers for classical breeding. We have shown that *R8* is present in at least five varieties from three different continents and in the Ma*R8* differential plant (which is identical to Black’s *R8* differential). So, *R8* donors for breeding are broadly available. Sequence analyses of the *R8* alleles revealed no significant *R8* allelic variation among the six sources. Therefore, it can be concluded that each of the six *R8* sources are equally potent for breeding.

It must be noted that the level of resistance provided by *R8* is highly dependent on the genetic background. In some backgrounds, the resistance level is sufficient to be detected using detached leaf assays, as is the case in Ma*R8* plants. But in F1 populations derived from Ma*R8,* resistance is only detectable in whole plant assays (Jo et al. 2011). Also in Sarpo Mira, late blight resistance can only be detected in whole plant assays (Orłowska et al. 2012). In F1 populations derived from Sarpo Mira, the *Rpi*-*smira2/R8*-mediated resistance was not apparent as a qualitative resistance, but rather was characterised as a quantitative resistance in whole plant/field conditions (Rietman et al. 2012). In our current study, where we transformed the *R8* gene to the genetic background of Desiree, the level of resistance in whole plant/climate chamber conditions ranged from intermediate resistance, characterised by expanding HR lesions, to full resistance (Fig. 2a; Table 1). This suggests that in some transgenic events, the *R8* gene is better expressed than in others. It remains to be established if the difference in resistance is correlated with T-DNA copy number and/or *R8* transcript level, as was observed for *RB* transgenic events (Bradeen et al. 2009; Kramer et al. 2009). Both in classical and GM breeding strategies, the resistance levels of the introduced *R* genes must be closely controlled. This is increasingly difficult with the number of *R* genes that are introduced, as late blight resistance assays can often not clearly measure the functional expression of all introduced *R* genes. We found that the response of *Rpi*-*sto1*, a close relative of RB, to its cognate Avr (IPI-O; Pieterse et al. 1994; Vleeshouwers et al. 2008) is strictly correlated with the level of resistance in transgenic plants (Zhu et al. 2013). Thus, Avr responsiveness is the most preferred tool to validate functional expression of *R* genes in stacks, both in classical and GM breeding. It remains, however, to be established how Avr8 response correlates with *R8* mediated late blight resistance levels.

## Electronic supplementary material

Below is the link to the electronic supplementary material.

**Figure S1.** Pulsed Field electrophoresis of randomly selected BAC clones from the second Ma*R8* library, digested with *Not*I. (PPTX 425 kb)

**Table S1.** Molecular markers for finemapping of *R8*. (DOC 49 kb)

**Table S2.** Primers used for subcloning of RGAs. (DOC 47 kb)

**Table S3.** Percentages of identity between R proteins from *Solanaceae*. (XLSX 10 kb)
