# Supplementary figures and images for: The Solanum demissumR8 late blight resistance gene is an Sw-5 homologue that has been deployed worldwide in late blight resistant varieties
Source: Theor Appl Genet. 2016 Jun 17;129:1785–96. doi: 10.1007/s00122-016-2740-0 (PMC4983296; doi:10.1007/s00122-016-2740-0)

## Slide 1
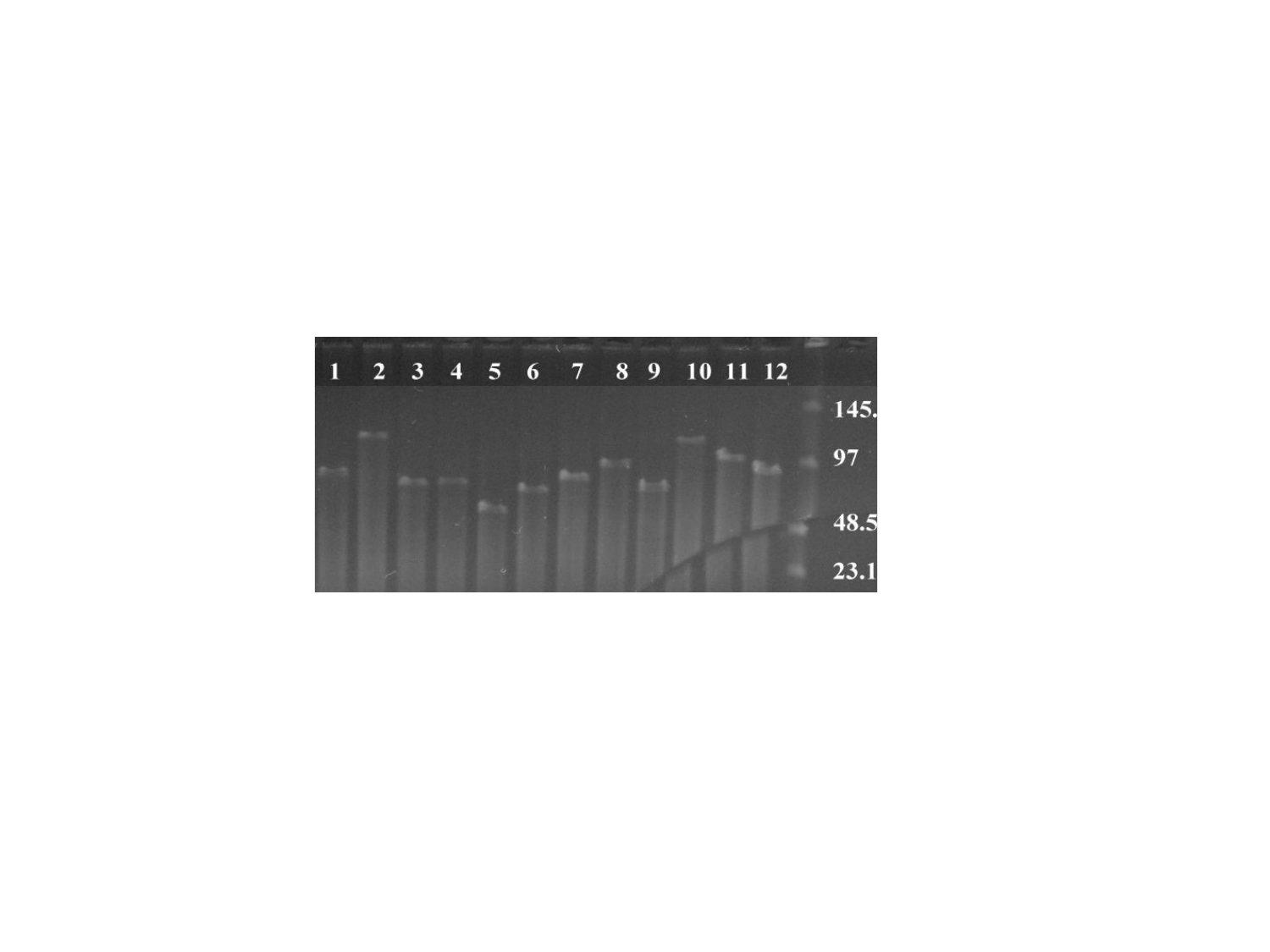

Supplement: Supplementary file 1 — Figure S1. Pulsed Field electrophoresis of randomly selected BAC clones from the second MaR8 library, digested with NotI. (PPTX 425 kb) [file 122_2016_2740_MOESM1_ESM.pptx]
