# Supplementary material for: The Solanum demissumR8 late blight resistance gene is an Sw-5 homologue that has been deployed worldwide in late blight resistant varieties
Source: Theor Appl Genet. 2016 Jun 17;129:1785–96. doi: 10.1007/s00122-016-2740-0 (PMC4983296; doi:10.1007/s00122-016-2740-0)
Supplement: Supplementary file 2 — Table S1. Molecular markers for finemapping of R8. (DOC 49 kb) [file 122_2016_2740_MOESM2_ESM.doc]

Table S1. Molecular markers used for *R8* finemapping

| Primer name | Primer sequence | Marker type | Amplicon size (bp) | Ta (°C) | Restriction enzyme | Reference |
| --- | --- | --- | --- | --- | --- | --- |
| 184_81F | CCACCGTATGCTCCGCCGTC | CAPS | 790 | 58 | *Rsa*I | Jo et al. 2011 |
| 184_81R | GTTCCACTTAGCCTTGTCTTGCTCA |  |  |  |  |  |
| CDP^Hero^3 | RRAGATTCAGCCATKGARATTAAGAAA | CDP | 501 | 55 | *Hae*III | Jo et al. 2011 |
| Adaptor primer | ACTCGATTCTCAACCCGAAAG |  |  |  |  |  |
| At5g06360_2F | AACAAGTAGTGCACGGACAAAAAC | CAPS | 984 | 58 | *Fsp*BI | This study |
| At5g06360_2R | AATGATGGCCTGATGGGTGAT |  |  |  |  |  |
| R8-3E3_10F | TTTGCCCCTGCTGAGAAGAATCC | SCAR | 2182 | 65 | na | This study |
| R8-3E3_10R | TCCACGTGCCAAAATAAACCAACC |  |  |  |  |  |
| R8-3E3_5F | TCAATGCAGCGCTTTAGGAT | HRM | 170 | 60 | na | This study |
| R8-3E3_5R | TGGACATGCAATTTTGACTTCTT |  |  |  |  |  |
| R8-2E2_1F | CAAGTTCCTGACCATTACAAAAGT | CAPS | 200 | 58 | *Hin*1II | This study |
| R8-2E2_1R | CAACGATGGTACCGATGGAT |  |  |  |  |  |
| R8-6A5F_3F | ACCCGCCAAATGAAACAACCAATG | CAPS | 1200 | 60 | *HpyCH*4IV | This study |
| R8-6A5F_3R | TTATACGGGGAAAGATGGGGTGTG |  |  |  |  |  |

Ta: Annealing temperature; CAPS: cleaved amplified polymorphism; CDP: cluster directed profiling; HRM: high resolution melting point polymorphism; na: not applicable
