# Supplementary material for: The Solanum demissumR8 late blight resistance gene is an Sw-5 homologue that has been deployed worldwide in late blight resistant varieties
Source: Theor Appl Genet. 2016 Jun 17;129:1785–96. doi: 10.1007/s00122-016-2740-0 (PMC4983296; doi:10.1007/s00122-016-2740-0)
Supplement: Supplementary file 3 — Table S2. Primers used for subcloning of RGAs. (DOC 47 kb) [file 122_2016_2740_MOESM3_ESM.doc]

Table S2: Primers used for RGA amplification and (sub)cloning

| Target | Orientation | Restriction site | Tm (°C) | Sequence (5’...3’) | Goal |
| --- | --- | --- | --- | --- | --- |
| RGA0.20 | F | *Srf*I | 63 | catgcccgggcAAAACTTTCACGCACCCATAGGA | RGA subcloning |
|  | R | *Sbf*I |  | gcacctgcaggAACAAGAGATGAATTAAGTCGGTAGC |  |
| R8 | F | AbsI | 60 | agtcctcgaggAAAACTTTCACGCACCCATAGGA | Allele mining |
|  | R | SrfI |  | catgcccgggcAACAAGAGATGAATTAAGTCGGTAGC |  |
| RGA0.21 | F | *Srf*I | 65 | catgcccgggcTTTGTATTATGATTGGCCCTGTTCTGA | RGA subcloning |
|  | R | *Sbf*I |  | gcacctgcaggCTGCGAGTATTGGTGGCTGACAT |  |
| RGA1.0 | F | *Srf*I | 65 | catgcccgggcCAACAATGCGGCGCTTTAGGA | RGA subcloning |
|  | R | *Sbf*I |  | gcacctgcaggTGGTGCACTTGCCTGGACTTTA |  |
| RGA1.1 | F | *Srf*I | 62 | catgcccgggcCCTCCATTTTCCATTAAGTCTTGC | RGA subcloning |
|  | R | *Sbf*I |  | gcacctgcaggAATGGTTTCATCAATGAATCTTTC |  |
| RGA1.2 | F | *Asc*I | 65 | catggcgcgccTATTAAACAGGAAACACAAAAGCAGTCA | RGA subcloning |
|  | R | *Xma*I |  | catgcccgggcTTCCTTGCAAAAATCTCACTCACTATG |  |
| RGA3.1 | F | *Srf*I | 62 | catgcccgggcCACCTAACTGATTTGCTTC | RGA subcloning |
|  | R | *Sbf*I |  | gcacctgcaggTCAAACTAAGACACTTAAATTA |  |
| RGA3.2 | F | *Sbf*I | 65 | gcacctgcaggTCTTGTGGTTGTCTTGGTAGCAGGAG | RGA subcloning |
|  | R | *Xma*I |  | catgcccgggcGTAGAGAAAAAGGAGAAAGCACAGA |  |
